# Supplementary material for: Are carbon emissions trading and green financial instruments synergistic? -Comprehensive quantitative research based on content analysis
Source: PLoS One. 2024 Mar 7;19(3):e0298601. doi: 10.1371/journal.pone.0298601 (PMC10919601; doi:10.1371/journal.pone.0298601)
Supplement: S1 Data — (ZIP) [file pone.0298601.s001.zip › Data/地级市Z_F工作报告.zip!/╔╜╢1⁄2/▒⌡╓▌╩╨/└┤╫╘╣1⁄2╓┌║┼í╛┬φ┐╦╩2╛▌═°í┐_2016.docx]

各位代表:

现在，我代表市人民政府向大会报告工作，请予审议，并请市政协委员和其他列席会议的同志提出意见。

一、“十二五”时期工作回顾

过去的五年，是滨州发展史上极不平凡的五年。面对新情况、新问题，全市上下深入学习贯彻习近平总书记系列重要讲话精神，认真落实中央和省决策部署，在中共滨州市委的坚强领导下，开拓创新，攻坚克难，实干兴业，经济和社会各项事业都取得了新成就。

（一）经济综合实力跃上新台阶。地区生产总值由2010年的1552亿元增长到2355亿元，增长56.9%，年均增长9.4%；一般公共预算收入由104亿元增长到204.1亿元，增长96.3%，年均增长14.4%；固定资产投资由886亿元增长到1990亿元，增长124.6%，年均增长17.6%；规模以上工业增加值增长74.6%，年均增长11.8%；社会消费品零售总额由451亿元增长到814亿元，增长80.5%，年均增长12.5%；进出口总额由51亿美元增长到81.4亿美元，增长59.8%，年均增长9.8%；金融机构本外币存贷款余额双双突破2000亿元，分别由1066亿元、1068亿元增长到2375亿元、2095亿元，年均分别增长17.4%、14.4%。

（二）民生福祉得到较大提升。累计民生支出1020亿元，是“十一五”时期的2.6倍。城镇、农村居民人均可支配收入分别由19686元、7194元增长到28388元、12727元，同口径相比增长68.6%、91.9%，年均增长11%、13.9%。城镇新增就业32.2万人次，农村劳动力转移就业64.2万人次。农村贫困人口由30万人减少至11万人。居民基本养老保险基础养老金由每人每月55元提高到85元，企业最低工资、退休人员养老金和城乡低保标准稳步提高。新建标准化村级卫生室910处，在全省率先实现乡镇卫生院住院“起付线以上全报销”。新开通公交线路191条，基本实现城乡公交一体化。累计投资32亿元改善基础教育办学条件，新建改扩建校舍260万平方米。购置校车867辆，在全省率先实现校车全覆盖。开工建设保障性住房3.2万套，改造棚户区187个、501万平方米。新建农村住房10万户，改造危房1.4万户。实施市城区环境综合整治，新建改造农贸市场26处，整治老旧小区494个，改建旱厕464处，拆除乱搭乱建近2万处，群众生活环境质量明显提升。市社会养老服务中心落成启用，完成83处乡镇敬老院旱厕改造，养老服务水平不断提高。设立了“特困家庭救助基金”，募集9719万元，已救助799户。2014年以来，连续两年全省民政考核群众满意度居全省第一位。文化、体育事业蓬勃发展，市“一馆三中心”、“文化三馆”、体育健身广场逐步投入使用。

（三）重大基础设施建设实现历史性突破。滨州港累计投资69亿元，其中“十二五”期间投资60亿元， 2个3万吨级码头正式通航，打开了滨州对外开放的海上通道。德大铁路投资29.3亿元，去年9月开通到北京等客运线路，彻底结束了滨州不通客运列车的历史。滨德高速建成通车。济滨东高速滨州段累计投资33亿元，完成总工程量的67%。秦滨高速埕口至沾化段、长深高速高青至广饶段、滨港铁路二期开工建设，黄大铁路、济青高铁加快推进，市城区依托黄河大堤实施南外环扩建工程获得国家黄河水利委员会批复。

（四）结构调整步伐更加坚实有力。三次产业结构由10.0：54.6：35.4调整为9.3：48.8：41.9。粮食连年增收，市级以上农业龙头企业由240家增长到372家，“三品”认证由275个增长到347个。持续实施林水会战，完成水利工程1300项、合格造林103万亩。黄河三角洲国家农业科技园区、滨城国家农业科技园区、沿黄生态高效现代农业示范区加快建设，“渤海粮仓”示范区面积发展到25万亩。工业发展提质增效，规模以上工业主营业务收入由4269亿元增长到7999亿元，年均增长13.4%。完成工业技改投入2699亿元，年均增长22.4%。高新技术产业产值由835亿元增长到1883亿元，年均增长17.7%。新增省级以上企业技术中心51家、山东名牌产品13个、中国驰名商标10件。魏桥集团销售收入达到3385亿元，居世界500强第234位。愉悦家纺荣获国家科学技术进步二等奖。积极化解过剩产能，钢铁、电解铝产能列入工信部符合行业规范条件名单。现代服务业加快发展，服务业增加值由549亿元增长到988亿元，年均增长9.2%。电子商务年交易额突破1000亿元，中国淘宝村达到16个。

（五）生态美丽幸福新滨州建设取得新成绩。市城区基础设施累计投资66.9亿元，比“十一五”增长67.6%，修改建道路290公里、桥梁51座，打通城市道路18条。盘活了备受社会关注的中海航母、国际会展中心等一批闲置资产。对新立河进行集中整治，打造了秦皇河公园等一批城市景观，成功创建国家园林城市。城镇化率由45.7%提高到54.6%。新农村建设步伐加快，累计建设农村新型社区277个。农村电网投资22亿元，完成8批次改造升级。自2013年开始，累计投入80亿元，完成4505个村庄、1.5万公里的农村道路“三通”任务，净化村庄5240个，美化村庄4973个，城乡环卫一体化实现全覆盖，去年群众满意度列全省第三位。节能减排成效明显，二氧化硫、化学需氧量排放量分别下降17%、13%，去年“蓝天白云”天数达到195天，比上年增加19天。两次代表山东省迎接国家海河流域检查，均获第一名。安全生产监管、食品药品安全工作不断加强。

（六）改革开放进一步深化。市县政府机构改革、国有企业改制、土地承包经营权确权登记颁证工作基本完成。财税体制改革、农村信用社银行化改革深入推进。连续举办5届资本（银企）对接大会，融资6591亿元。医药卫生体制改革累计投入84.8亿元，县级公立医院综合改革扎实推进。民营经济快速发展，新增私营企业2.6万家、个体工商户9.4万户。开放型经济更加活跃，引进世界500强企业25家，实际到账外资26.8亿美元，境内外上市公司达到15家。全面实施人才强市战略，引进各类高层次人才821人。实施境外投资项目46个，15家企业到国外开发资源、投资设厂、开展并购。鲁中运达保税物流中心开关运营，“滨新欧”国际货运班列正式开通。沾化撤县设区，邹平经济技术开发区、滨州经济技术开发区先后上升为国家级开发区。对口支援重庆奉节县、青海祁连县工作成效明显。

（七）法治政府建设取得明显成效。坚持廉洁勤政、依法行政，加大简政放权力度，市直行政许可事项压减至315项，非行政许可事项全部取消，行政审批事项目录清单、市级权力清单、政府责任清单编制完成，并向社会公布。深入开展党的群众路线教育实践活动、“三严三实”专题教育，查处违反中央八项规定精神问题476起，市级机关清退办公用房5.9万平方米，集中调整安置市直单位20个。畅通群众诉求渠道，受理市长公开电话36.7万个、市长信箱来信6300封。主动接受人大监督和政协民主监督，办理人大代表建议446件、政协提案1483件，办结率均达到100%。荣获首届中国“阳光政府奖”，市公共资源交易中心获评中国公共采购十佳最具公信力机构。

各位代表，刚刚过去的2015年，是“十二五”时期的收官之年，更是滨州发展史上具有里程碑意义的关键一年。一年来，面对经济下行压力的巨大考验，我们克服种种困难，经济实现了协调平稳发展，质量效益得到有效提升。一年来，我们咬定青山，实现了港口通航、铁路通车，期盼多年的一批高速、铁路项目陆续开工，重大基础设施建设取得历史性突破。一年来，我们坚持奋勇争先，国家卫生城复审、国家海河流域环境检查、入选全国文明城市提名城市，一批难度大、要求高的硬性任务高标准完成。一年来，我们坚持民生优先，实施农村道路“三通”、农贸市场改造等老城区提升、新立河治理等工程，办成了一批投资大、惠及面广的实事好事。一年来，我们坚持积极争取协调，组建滨州农商行获得银监会正式批准，依托黄河大堤建设城市南外环获得国家批准，电解铝、钢铁产能全面纳入国家统计范围，国家的基金投资、政策性银行资金争取名列全省前茅。一年来，我们坚定不移实施“走出去”战略，魏桥集团成功在几内亚开矿，建成了国家重要原材料基地，一批企业引进来、走出去不断取得新成效。全年地区生产总值增长7.1%，一般公共预算收入增长9.1%，固定资产投资增长13.8%，城镇和农村居民人均可支配收入分别增长7.5%和8.8%。2015年主要目标的实现，标志着“十二五”任务的顺利完成。

各位代表，五年的探索和实践，我们深刻认识到：做好政府工作，必须坚决贯彻中央、省决策部署，勤政务实，创造性地开展工作；必须坚持民生为先，实打实地为民办实事、办好事；必须坚持发展第一要务，一张蓝图干到底，咬定目标不放松；必须坚持依法行政，自觉运用法治思维和法治方式破解各种难题；必须坚决服从市委领导，自觉接受人大监督，加强多党合作和政协民主协商，尊重人民主体地位。只要坚持这些好的经验做法，就一定能够赢得广大群众的拥护支持，凝聚起建设生态美丽幸福新滨州的强大合力。

各位代表，过去五年的进步和变化，得益于省委、省政府的方向引领和深切关怀，得益于中共滨州市委的科学谋划和统揽指挥，得益于市人大、市政协和社会各界的大力支持和共同努力，凝聚着全市人民的智慧和汗水。在此，我代表市人民政府，向全市人民、各界人士、驻滨部队官兵和公安干警，向所有关心支持滨州发展的海内外朋友，致以崇高的敬意和衷心的感谢！

各位代表，在看到成绩的同时，我们也清醒地认识到，经济社会发展中还存在不少问题。主要是：经济结构不合理、不协调、不可持续问题比较突出，企业整体创新能力不强，产业层次低，资源、环境约束和节能减排、安全生产压力大；招商引资力度不大，引进项目的投资体量小，科技含量、产出效益不高；加强金融监管、提高服务能力、防范风险还有大量工作要做；城乡差别、区域差别比较明显，基础承载力不强；部分群众生活困难，脱贫攻坚任务艰巨，教育、养老、医疗卫生、社会保障等领域还存在不少“短板”；政府机关和工作人员还存在法纪观念、服务能力不强，办事效率不高、不严不实等问题。对这些问题，我们将认真对待，努力加以解决。

二、“十三五”时期的主要目标和重点任务

今后五年，是全面建成小康社会的决胜阶段。我们既面临诸多风险挑战，也面临难得的历史机遇。“十三五”时期全市经济社会发展的总体要求是：深入贯彻习近平总书记系列重要讲话精神，按照“四个全面”战略布局，积极适应和引领经济发展新常态，坚持以人民为中心的发展思想，把创新、协调、绿色、开放、共享五大发展理念贯穿于发展全过程，坚持“六个更加注重”，努力推动产业迈向更高端、基础建设更完善、生态环境更优美、改革开放更深入、社会局面更和谐、人民生活更幸福，保持经济社会持续健康发展，确保提前全面建成小康社会。

“十三五”时期的经济社会发展主要目标是：

——经济保持稳定增长。地区生产总值年均增长7.5%左右，城乡居民人均可支配收入年均增长7.6%左右，一般公共预算收入年均增长8%左右，固定资产投资年均增长13%左右，提前实现经济总量和城乡居民收入比2010年翻一番。坚持又好又快发展，努力缩小与先进地区差距。

——产业逐步迈向中高端。规模以上工业企业主营业务收入达到1.1万亿元，高新技术产业产值占比明显提升。服务业占GDP比重达到46%。创新型城市建设取得积极进展，全社会研发经费占比提高到2.8%。

——人民生活水平显著提高。基本公共服务均等化稳步提升，就业得到充分保障，收入分配差距持续缩小，健康水平不断提高。新型城镇化、城乡一体化进程加快，常住和户籍人口城镇化率分别达到60%和50%左右。

——生态环境更加优美宜居。经济社会发展方式更加优化，全面完成省下达节能减排目标。城乡面貌明显改善，美丽乡村建设更加深入，2017年全面完成农村改厕。林木绿化率达到27%，环境空气质量在2010年基础上改善50%左右。

——社会事业蓬勃发展。法治政府基本建成，人民民主更加健全。重要领域和关键环节改革取得积极成果。文化、医疗卫生事业加快发展，教育投入大幅增长，人民群众精神文化生活更加丰富。社会保障体系更加健全，食品药品安全、交通安全、生产安全监管体系更加完善，社会更加和谐稳定。

要实现上述奋斗目标，必须全面贯彻五大发展理念，着力推进全市经济社会又好又快发展。

（一）深刻把握创新发展内涵要义，加速发展动力转换。面对新常态、适应新常态、引领新常态，必须把创新摆在发展的核心位置。要把发展基点放在创新上，把大众创业、万众创新融入各领域各环节，培育发展新动力。毫不动摇地支持引导非公有制经济发展，全力培育新的经济增长点。积极拓展发展新空间，加大北部沿海开发建设力度，完善交通、水利、电力等基础设施网络。实施“互联网+”行动计划，大力发展网络经济。发挥科技创新的引领作用，把增强技术实力作为构建产业新体系的战略支点，推动数控技术和智能装备的广泛应用，激发产业创新发展的能力水平。深入推进人才强市战略，瞄准“千人计划”、“泰山学者”、国外高端人才和管理团队，加大引进力度。改革影响发展的体制机制，提高政府效能，进一步激发市场活力和创造力。

（二）深刻把握协调发展内涵要义，着力补齐发展短板。认真分析城乡差距、南北差距、基础设施等差距，进一步加大工作力度，促进区域城乡协调发展。依托滨州港，发展临港产业，推动北部沿海加快开发建设。提高中心城区承载能力，支持惠民、阳信、无棣加快工业化步伐，提升增量，带动发展；促进博兴、邹平优化经济结构，提高质量效益。推进以人为核心的新型城镇化，统筹城乡基础设施建设，推动养老、医疗、金融网点等服务设施向农村延伸。加快高速公路建设，实现“县县通高速”目标。推进高铁和沿海高等级公路、德大铁路动车化建设，打造与环渤海、京津冀、省会城市群互联互通的区域性交通枢纽。坚持物质文明和精神文明协调发展，弘扬社会主义核心价值观，加快文化改革发展，加强思想道德和社会诚信建设，深入开展全国文明城市创建活动，促进经济社会协调发展。

（三）深刻把握绿色发展内涵要义，实现发展与生态共赢。统筹抓好节约资源和环境保护工作，坚持走可持续发展之路。树立节约集约循环利用的资源观，推动建立绿色低碳循环发展的产业体系。牢固树立底线思维，把大气污染治理放在突出位置，减少雾霾天气发生，实施最严格的环境保护制度。做好主要污染物总量减排，坚持能耗和水资源消耗、建设用地等总量和强度“双控”，按计划完成超低排放改造。落实最严格的水资源管理制度，加强对徒骇河、马颊河、漳卫新河等重点流域的水污染防治，推动水生态文明城市建设。抓好重点区域治理，推动以大气、水和土壤为重点的污染综合治理，强化农业面源污染防治。加快电网建设，实施“气化滨州”、煤改气等能源替代工程，不断优化能源结构，推动低碳循环发展。

（四）深刻把握开放发展内涵要义，推动高水平双向开放。加快对外贸易优化升级，提升传统优势产品竞争力，巩固出口市场份额。加大招商引资力度，认真研究引进优质资源、先进设备、高端人才和管理经验，特别要注重引进世界500强和各类大型企业集团。要积极走出去，鼓励企业参与境外资源开发利用，实施兼并收购，开拓国际市场。积极融入“一带一路”、中韩自贸区建设，融入环渤海地区合作发展，全面参与京津冀协同发展，拓展对外开放空间。

（五）深刻把握共享发展内涵要义，坚持以人民为中心的发展思想。发展依靠人民、发展为了人民、发展成果由人民共享。重点实施扶贫攻坚战略，确保2017年农村贫困人口全面脱贫。合理布局教育资源，集中解决好“大班额”问题，推动基础教育均衡提升，提高教育教学质量。科学配置公共医疗资源，基本建成覆盖城乡的基本医疗卫生制度。实施积极的就业政策，继续开展农民工“三项行动计划”。实施全民参保登记计划和城乡低保一体化，建立更加公平的社会保障制度。积极稳妥地实施全面两孩政策，继续降低出生人口性别比，促进人口均衡发展。推动“食安滨州”建设，保障群众舌尖上的安全。抓好安全发展，确保各领域安全形势总体稳定。加快建设覆盖城乡的公共法律服务体系。加强社会治安综合治理，严厉打击违法犯罪活动，维护社会和谐稳定。

各位代表，展望“十三五”，我们满怀期待，充满信心。只要我们主动适应新常态，迎难而上，合力共为，敢于担当，全面建成小康社会的宏伟目标就一定能够实现！

三、2016年工作安排

今年是全面建成小康社会决胜阶段的开局之年，经济社会发展的主要预期目标是：地区生产总值增长7.5%左右，一般公共预算收入增长8%左右，规模以上工业增加值增长8%左右，固定资产投资增长13%左右，社会消费品零售总额增长10%左右，进出口保持稳定增长。城镇和农村居民人均可支配收入分别增长8%左右和9%左右，城镇登记失业率控制在3.6%以内。全面完成年度节能减排约束性指标。

实现今年的目标任务，我们要继续坚持稳中求进工作总基调，扎实推进供给侧结构性改革，全面落实去产能、去库存、去杠杆、降成本、补短板“五大任务”，努力提高发展的质量效益。

（一）坚持稳中求进，抓好工业经济。稳中求进是今年经济工作的总基调，工业是滨州经济的主要支撑，能否实现稳中求进，工业经济责任重大。要抓好稳增长，稳增长的重点是现有骨干企业，要全方位支持优势骨干企业加快发展，抓好稳增长政策落实，强化金融对实体经济的支持，为实体经济让利。在控制新增能耗、逐步缩减排放的前提下，依托魏桥、京博、西王、滨化、北汽集团等骨干龙头企业，逐步打造5000亿级高端铝产业集群、2000亿级新型化工产业集群、1500亿级粮食加工产业集群、1300亿级纺织产业集群、全球高端汽车轮毂制造中心和汽车轻量化材料基地，通过重点企业带动，确保全市经济稳定增长。要抓好工业经济质量提升，一是抓好供给侧结构性改革。围绕妨碍发展的制度性障碍，进一步深化制度变革。政府各部门要为企业发展提供更加优质的服务，充分调动企业家的积极性、主动性和创造性。实施质量强市和品牌战略，支持鼓励企业主导或参与制定国家和国际标准，提高产品质量水平。要围绕市场需求开发适销对路产品，搞好个性开发、差异化服务，把产品做精做强，努力打造百年企业，推动实现由要素驱动向创新驱动转换，由价格竞争向技术服务品牌竞争提升，由高碳式向低碳式转型。认真落实“去产能”要求，对产能过剩行业一律不再新增，对落后产能坚决淘汰。二是加快创新转型，实现整体素质提升。引导骨干企业带头融入“中国制造2025”，鼓励企业与国内外科研机构对接合作，搞好技术、设备、人才、管理、市场的联合、引进，“借梯上楼”、“借船出海”，努力提高企业核心竞争力。三是面对经济下行压力下市场“重新洗牌”和原材料价格低廉的时机，抓好国家和省重点项目、重点基金争取，千方百计加大投资，进一步优化结构，搞好技术改造、装备更新，拉长产业链，提高产品档次，巩固老市场，开拓新市场。

（二）大力发展现代农业。提高粮食综合生产能力，深化开展高产创建、“渤海粮仓”、高标准农田建设工程，抓好70万亩粮食生产“十统一”社会化服务。加强中低产田改造提升和基本农田保护，做好永久基本农田划定工作。改善农业生产条件，抓好引黄工程、平原水库和雨洪水资源利用工程建设，全面完成南水北调配套工程。加快小农水重点县工程建设，抓好引黄灌渠清淤管护，推进水利灌排渠系向边远地块延伸，确保黄河来水引得出、存得住、用得好。推广阳信县小型农田节水灌溉模式，利用市场化手段兴办水利事业。围绕机种机收、秸秆还田、饲料青贮，进一步提高农业机械化、信息化水平，做好良种繁育推广。优化农业结构，抓住增加农民收入这个关键，利用生物、信息技术改造提升传统农业，做强蔬菜、林果、畜牧、渔业等优势产业。深化对接京津冀、省会城市群的“米袋子”、“菜篮子”、“果盘子”工程，加快沿黄生态高效现代农业示范区建设，着力打造高新区现代农业示范园等一批特色园区。因地制宜发展青贮玉米、优质牧草，大力发展畜牧业。推进冬枣、鸭梨等优势林果品牌打造、质量提升。建设“海上粮仓”，打造沿海水产高效健康养殖产业带。开展农产品质量安全县创建，保障群众食品安全。推进农业经营方式创新，加快农业龙头企业升级改造、链条延伸，带动一二三产融合发展。放大省级以上农业园区示范作用，积极发展农民合作社和家庭农场，推进土地流转，发展适度规模经营。抓好秸秆禁烧，推动农业绿色发展。

（三）壮大提升现代服务业。拓展新领域，催生新业态，推动生产性服务业向专业化和价值链高端延伸、生活性服务业向精细和高品质转变。抓好物流园区布局建设，依托港口、铁路物流潜力，积极对接省会城市群经济圈大型企业货运需求，抓好沿线布点，重点谋划建设好现代物流园区。加快发展电子商务，推进电子商务进农村、进社区，引导电商业户集聚发展，培育支持一批电商龙头企业、示范园区。鼓励各类经营主体申办进出口经营权，开展跨境电子商务业务。强化重点项目带动，加快万达广场、颐高国际等重点项目建设进度，争取新引进落地一批大项目。积极发展旅游产业，完善提升基础设施，促进历史文化、黄河风情、乡村民俗、生态自然与旅游深度融合，策划打造精品线路。精心办好第二届中国古村镇大会，大力发展乡村旅游。

（四）加快重大基础设施建设。加快港口建设，完成滨州港3万吨航道和海港区防波堤工程建设，增加2×3万吨级散杂货码头吞吐量。争取3万吨级液化码头主体完工，加快码头泊位建设，尽快增加新的泊位运行。完成套尔河港区3000吨级航道及防波挡沙堤工程前期工作。加快铁路建设，推进滨港铁路二期、黄大铁路、济青高铁建设，争取山东“市市通高铁”计划在滨州尽快实施。加快高速公路建设，全力推进济滨东高速建设，确保年内通车。加快秦滨高速埕口至沾化段、长深高速高青至广饶段和济青高速改扩建工程。加快城乡公路建设，依托黄河大堤加快南外环扩建，确保年内达到通车条件。加快国省道干线公路改造，确保省道寿济路邹平段建成通车、滨惠大道全面贯通、市城区东外环北延工程主体完工。加快沿海高等级公路前期工作，争取早日开工。全面完成国省道和农村公路安全生命防护工程项目和延伸，提升安全通畅水平。加快能源项目建设，抓好国电博兴2×100万千瓦机组项目开工建设，做好华能沾化2×100万千瓦机组项目前期工作。深入推进“气化滨州”建设，研究推进天然气进农村工程，逐步健全天然气输配管网。

（五）积极推进城乡建设。提升城市规划水平，高标准编制完成《滨州市城市总体规划（2016-2030）》，积极做好城市综合交通体系、地下综合管廊、北外环河改造等重点规划。完善城区基础设施，加大基础设施投资，实施道路新建改造、污水管网、公园设施改造、供热等工程。启动黄河四桥前期工作，推进海绵城市建设试点，积极开展黑臭水体治理和城区雨污分流改造。完善“四环五海”生态水系，推进河湖水系连通工程。完成城南引黄沉沙池建设，实现清水入城。建立完善城市管理长效机制，全力创建全国文明城市。加大住房保障力度，落实“去库存”任务，力争消化存量商品房50万平方米。完成棚户区改造9223户，实施市城区老旧小区改造，改善居民居住条件。加快推进市民化进程，实现7万农业转移人口市民化，2.7万城中村、城边村居民完全市民化。加快各具特色的小城镇建设，提高产业支撑能力，为农业转移人口创造就业条件。改善农村人居环境，围绕建设美丽宜居村庄，改造农村厕所46.4万户，优先做好老年人、残疾人、困难家庭改厕工作。加大农村危房改造力度，力争完成2000户。推进农村电网改造，新建、改造线路2521公里。加大农村生活污水处理力度，试点建设村庄小型污水处理设施。

（六）深入扩大对外开放。集中精力抓好招商引资，全面梳理完善政策，重点围绕电解铝、化工、纺织、机械等产业，围绕港口开发建设，抓住京津冀协同发展重大机遇，组织系列招商活动，特别要抓好世界500强的合作引进。推动外贸平稳增长，全面落实外贸稳增长政策，抓好纺织企业棉花配额和新增企业进口原油指标争取，稳定铝矿砂、大豆等重点进口产品。大力培育出口支柱企业，新增备案企业200家以上。加强平台载体建设，抓好进出口贸易综合服务平台和外贸基地建设。推动鲁中运达保税物流中心规范运营，滨州港一类开放口岸申报取得实质性进展。创新开发区体制机制，推动转型升级。加快“走出去”步伐，发挥企业主体地位，实施并购重组国外企业，抓好一批境外投资项目建设，加强国际产能合作，不断提高企业的国际化水平。

（七）加大环境保护力度。突出抓好大气和水体污染治理，启动大气污染防治二期行动计划，对10万千瓦及以上燃煤机组实施超低排放改造，力争2017年全面完成。加强重污染天气应急应对，努力增加“蓝天白云”天数。利用市场机制提高污水处理质量，实行“优水优价、阶梯水价”。完成8条重点河流沿线污染源排查和8个工业园“一企一管”改造，深入推进全流域治理。加大节能减排力度，严格煤炭消耗量控制、脱硝设施运行监管，加快关停违规小火电，完成410个违规建设项目清理整顿，努力消化新增排放量。加快生态工程建设，深入推进全国水生态文明城市创建，确保通过水利部中期评估。推进湿地公园建设，加强近岸海域环境保护。严格环保执法，健全完善环境保护督查巡视制度，深入开展“零点行动”，消除监管盲区和空白时段，公开曝光环境违法案件，严打环境违法犯罪行为，努力打造碧水蓝天的美好家园。

（八）全面深化各项改革。深化财税体制改革，推进全口径预算管理，所有县级以上使用财政资金的部门，全部依法依规公开预决算。围绕公共服务、基础设施等重点领域，扩大政府与社会资本合作。完成“营改增”改革任务，实行过渡期市对县区收入划分办法。深化国地税征管体制改革，形成联合办税新格局。深化金融体制改革，组建滨州农商行，全面完成农信社银行化改革，填补市级地方商业银行空白。加快推动直接融资，力争6家企业实现“新三板”挂牌。设立市级投资引导基金，组建市级资产管理有限公司，市县政府共同出资设立市级再担保公司。强化“诚信滨州”建设，严厉打击网络、金融诈骗和非法集资、恶意逃废债务等行为，化解担保圈、担保链风险，维护金融环境稳定。深化农业农村改革，抓好农村土地承包经营权确权登记颁证成果验收和应用，推动开展土地承包经营权、农民住房财产权抵押贷款，有效盘活农村集体资产和农民权益资源。深化供销合作社综合改革。强化涉农重点领域金融创新，扩大农业保险覆盖范围。深化商事制度改革，下放登记权限，最大限度为企业登记注册提供便利。全面落实“三证合一、一照一码”登记制度，加快电子营业执照和企业登记全程电子化。深化医药卫生体制改革，加快公立医院综合改革，巩固完善基本药物制度，推进分级诊疗试点。提高整体筹资标准，职工和居民医保政策范围内住院费用支付比例分别达到75%以上和70%左右。加快不动产登记改革，推进登记职责和机构整合，确保11月底前到位。加快事业单位分类改革，按单位类型明确经费来源，创新机构编制管理和公共服务提供方式，推动学校、医院、科研院所去行政化。稳妥推进公务用车制度改革，按照上级规定时限完成改革任务。

（九）着力改善人民生活。全力实施精准扶贫、精准脱贫，加大扶贫投入，市财政专项扶贫资金增加到5500万元。因户因人施策，实施精准帮扶。整合各类扶持政策，集中用于扶贫工作，做好兜底保障。严格考核落实，确保完成脱贫6万人以上。健全社会保障体系，巩固稳定就业形势，实现城镇新增就业3.5万人、农村劳动力转移就业5万人。扩大城乡居民养老、工伤、生育、失业保险覆盖面，完善城乡居民社保服务系统。提高城乡低保标准，确保不低于上年度城乡居民消费支出的25%和30%。创新养老服务模式，开展医养护一体化服务试点。完善医疗救助制度，扎实开展重特大疾病救助工作。完善困难残疾人生活补贴和重度残疾人护理补贴制度，两项补贴标准均提高到每人每月80元。大力发展社会事业，加大教育投入，突出抓好“大班额”改造，大力推进学前教育二期工程，完成足球进校园运动场地项目200个，实现县域内义务教育均衡发展，推动高中阶段教育协调发展，加快现代职业教育体系建设。积极推进基础教育综合改革，提升立德树人水平。大力发展文化事业，加快市文化中心大剧院建设，实现地面数字电视全覆盖。加强文化市场监管，扎实开展“扫黄打非”。推进“健康滨州”建设，优化调整市级医疗资源布局，强化食品药品安全监管。抓好竞技体育和全民健身，打造城区“15分钟健身圈”。深入开展双拥共建活动，增强军政军民团结。深化社会治理创新，加强社会治安，推进立体化防控体系建设，深入开展反恐和禁毒全民斗争。完善社区治理服务，建成100处规范化、示范型农村社区服务中心。狠抓安全生产，强化企业主体责任落实，深入开展大排查快整治严执法集中行动，坚决遏制重特大事故发生。

2016年，要完成好扶贫攻坚、农村改厕、改善办学条件等10件民生实事。

四、加强政府自身建设

新形势、新任务对政府工作提出了新要求。我们将坚持人民主体地位，勇担历史使命，顺应群众期待，认真履职尽责，对人民负责，让人民满意。

（一）切实加强政府系统党的建设。市政府党组要严格按照党章要求，在市委的集中统一领导下，围绕中心，突出重点，狠抓落实。政府系统广大党员干部，要增强看齐意识、核心意识，带头遵守党的政治纪律和政治规矩，在思想上、政治上、行动上同党中央保持高度一致。巩固党的群众路线教育实践活动成果，拓展“三严三实”专题教育实际成效，深入学习党章党规，学习习近平总书记系列重要讲话，做合格党员，打造信念坚定、为民服务、勤政务实的公务员队伍。

（二）严格要求，勇于担当。要主动适应新常态，在抓发展上体现新思路、新状态、新作为，以高度负责的担当，敢啃硬骨头，敢于涉险滩，坚定不移地推动各项改革。不断创新工作方式，建立合理的容错机制、及时的纠错机制和正常的免责制度。构建常态化督查机制，严格落实限期报告、调查复核、情况通报、责任追究等制度，着力克服庸懒散现象，认真解决不愿为、不会为、不敢为的问题。完善考核评价体系，对主动作为、做有成效的予以表彰，对不作为的严肃问责，鼓励各级干部想干事、敢干事、干成事，不断提高政府行政效率。

（三）依法规范行政行为。坚持依法治市，全面提升尊法学法守法用法意识，自觉运用法治思维和法治方式深化改革、推动发展。强化决策法定程序的刚性约束，坚持重大行政决策集体讨论决定。继续推进简政放权，加快省市县统一的政务服务平台、公共资源交易平台建设，坚决消除权力设租寻租空间。注重发挥律师、法律顾问和司法机关作用，通过协商调解和必要的法律程序解决问题。深入推进政务公开和政府信息公开，及时回应社会关切。自觉接受人大法律监督、工作监督和政协民主监督，重视舆论监督、社会监督。全面落实党的知识分子、民族、宗教、侨务等政策。认真听取民主党派、工商联、无党派人士的意见建议。支持好工会、共青团、妇联等群团组织的工作。

（四）切实加强廉政建设。落实从严治党主体责任，严格执行中央八项规定，弛而不息纠正“四风”。健全公共资金、国有资产、国有资源和领导干部履行经济责任审计全覆盖制度，加强对重要领域、重点工程和关键环节的监督管理，着力从源头上预防和治理腐败。严控“三公”经费和一般性支出，降低行政成本，力戒铺张浪费。加强民生资金审计监管，严厉查处套取、挤占、挪用、截留等损害群众利益的问题。加大违纪违法案件查办力度，严肃查处发生在群众身边的腐败问题，努力实现干部清正、政府清廉、政治清明。

各位代表，宏伟蓝图已经绘就，新的征程任重道远。让我们紧密团结在以习近平同志为总书记的党中央周围，在中共滨州市委的坚强领导下，统一思想，锐意创新，求真务实，砥砺奋进，为全面建成小康社会、开创生态美丽幸福新滨州建设新局面而努力奋斗！
